# Body mass index can affect gastrointestinal and genitourinary toxicity in patients with prostate cancer treated with external beam radiation therapy

**DOI:** 10.3892/ol.2013.1658

**Published:** 2013-11-05

**Authors:** HIROSHI DOI, FUMIKO ISHIMARU, MASAO TANOOKA, HIROYUKI INOUE, SOICHI ODAWARA, YASUHIRO TAKADA, YASUE NIWA, MASAYUKI FUJIWARA, NORIHIKO KAMIKONYA, SHINGO YAMAMOTO, SHOZO HIROTA

**Affiliations:** 1 Department of Radiology, Hyogo College of Medicine, Nishinomiya, Hyogo 663-8501, Japan; 2 Department of Clinical Radiology, The Hospital of Hyogo College of Medicine, Nishinomiya, Hyogo 663-8501, Japan; 3 Department of Urology, Hyogo College of Medicine, Nishinomiya, Hyogo 663-8501, Japan

**Keywords:** prostate cancer, body mass index, obesity, radiotherapy, external beam radiation therapy

## Abstract

The aim of the present study was to determine the impact of obesity on radiation-induced gastrointestinal (GI) and genitourinary (GU) toxicity. The cases of 54 patients with prostate cancer, treated with three-dimensional conformal radiation therapy (RT), were reviewed. For each patient, the body mass index (BMI), distance between the prostate and rectum (D) on a computerised tomography scan and the dosimetric parameters of the rectum and bladder in the planning data of RT, were analyzed. GI and GU toxicity was assessed during and following RT. The results of the patients with a BMI of <25 (lower BMI) were compared with those of the patients with a BMI of ≥25 (higher BMI). The higher BMI group exhibited significantly lower doses of V_60_ and V_65_ in the rectum than the lower BMI group. No significant differences were found in D and bladder doses between the two groups. The incidence of acute GI and GU toxicity and late GI and GU toxicity were 41.7, 19.4, 35.3 and 5.7% in the lower BMI group, respectively, and 27.8, 27.8, 5.9 and 35.3% in the higher BMI group, respectively. In addition, a significant difference was found in the incidence of late GU toxicity between the two groups. Among patients who underwent RT for prostate cancer, those with higher BMIs had a tendency to show lower incidences of GI toxicity and higher incidences of GU toxicity than patients with lower BMIs. To conclude, an increased effort must be made to reduce rectal doses in patients with lower BMIs. Conversely, increased care for GU toxicity must be provided for overweight patients.

## Introduction

Although radiation therapy (RT) is a well-established option for treating patients with prostate cancer, gastrointestinal (GI) and genitourinary (GU) toxicity is a major complication of RT. Radiation-induced toxicity can manifest as acute effects that occur during or rapidly following RT, or as late effects that arise between a number of months and years later. GI and GU toxicity remain important dose-limiting factors for the use of RT in patients with prostate cancer and can affect the quality of life of patients who undergo RT (1–6). In addition, current treatment strategies for patients with GI and GU toxicity are often unsatisfactory and there exists no recommended standard treatments for such patients.

The prevalence of obesity is increasing worldwide, although, large ethnic differences exist. The body mass index (BMI) is a simple ratio of weight to height that is commonly used to classify overweight and obese adults. It is defined as a patient’s weight in kg divided by the square of the patient’s height in m^2^ (kg/m^2^). The World Health Organization categorizes individuals with BMIs of >25 kg/m^2^ as being overweight (7).

It has been previously reported that obesity is associated with the incidence of more aggressive, higher grade prostate cancer and greater prostate-specific antigen (PSA) failure rates following radical prostatectomy or external beam RT (EBRT) (8–14). However, Efstathiou *et al*(15) previously reported that BMI is not associated with PSA failure in males with prostate cancer treated with brachytherapy. To date, only a small number of studies have analyzed the impact of obesity on radiation-induced toxicity and the correlation between patient BMI and radiation-induced toxicity remains uncertain.

The aim of the present study was to assess the impact of BMI on radiation-induced GI and GU toxicity.

## Materials and methods

### Patient selection

A total of 254 patients with prostate cancer were treated with RT at the Hospital of Hyogo College of Medicine (Hyogo, Japan) between January 2007 and December 2009. All data used was obtained from the patient’s medical records via a retrospective review. Written informed consent was obtained from the patients.

Patients were eligible for the study if they showed histological evidence of prostate cancer and underwent curative therapy with three-dimensional conformal RT (3D-CRT) to treat clinically localized prostate cancer. Patients with bone metastases that showed complete responses to treatment were included in the present study. Data, including Gleason scores, serum PSA measurements, clinical stages and adverse events, were obtained from patient records.

The patients were classified into groups according to pretreatment risk using a modification of the D’Amico risk classification system (16). Patients with T4 prostate tumors, with nodal or distant metastases, were assigned to the high-risk group.

Data collected within two years of the initiation of RT, including height, weight and the results of CT scanning to measure abdominal adiposity, were also used. Among all the records, acute toxicities were assessed in the 54 patients of which all data were available.

Two patients with follow-up terms of <3 months were not included in the assessment of late toxicities. One patient with a rectal invasion due to a recurrence of prostate cancer was excluded from the assessment of late GI toxicity. Late GI and GU toxicity were assessed in the remaining 51 and 52 patients, respectively.

### Treatment

All patients were placed in the supine position and helically scanned on an Aquilion LB (Toshiba Corporation, Tokyo, Japan) computer tomography unit. For each patient, a planning computerised tomography (CT) scan of the entire pelvis from the lower-abdomen to below the ischial tuberosities was obtained at 5-mm intervals. Patients were required to urinate and excrete stool prior to the CT scan.

The CT data set was transferred to the FOCUS XiO™ (CMS Inc., St. Louis, MO, USA) treatment planning system to outline the volumes of interest.

The clinical target volume (CTV) of the prostate and seminal vesicles was calculated in the intermediate- and high-risk prostate cancer patients. For these patients, the CTV was expanded in three dimensions with a 1-cm margin, with the exception of the prostate-rectal interface, where a 0.5-cm margin was used to obtain the planning target volume (PTV). The RT plan was prescribed at the isocenter in the PTV.

The normal structures were outlined and considered to be solid organs, including the rectum, bladder and femoral heads. In addition, the rectum and bladder were reviewed and contoured a second time in all patients eligible for the present study. For each case, the rectum was contoured from the anal verge or ischial tuberosities (whichever was higher) to the sacroiliac joints or the rectosigmoid junction (whichever was lower) (17). The bladder was contoured from the apex to the dome.

The patients were treated using the 3D-CRT technique with 8 coplanar fields and 10 MV photons. The RT plan was delivered using Mevatron KD2/50 Primus (Toshiba Corporation) between 2007 and 2009 and Elekta Synergy (Elekta, Crawley, UK) from 2009 onward.

Androgen suppressive therapy was started prior to the initiation of RT and was routinely used during the term of RT, with the exception of one low-risk prostate cancer patient.

### Anthropometric measurements and dosimetric parameters

The abdominal circumferences and areas of subcutaneous and visceral fat were measured retrospectively from the CT images. The CT images were reconstructed at a 5–8-mm thickness. For each patient, the abdominal circumference and areas of fat were measured on single cross-sectional scans obtained at the umbilicus (18). All images were analyzed on the Virtual Place Advance Plus workstation (AZE Ltd., Tokyo, Japan). The areas of subcutaneous and visceral fat were obtained by automatic planimetry. First, the subcutaneous and visceral fat were automatically defined. Subcutaneous fat was defined as the extraperitoneal fat between the skin and muscles and the intraperitoneal fat was defined as visceral fat. The border was manually corrected. Next, the areas of visceral and subcutaneous fat were automatically calculated from the mean value and standard deviation of the Hounsfield unit values in the fat tissue.

For each patient, the distance between the prostate and the rectum (D) from the anterior wall of the rectum to the superior margin of the prostate was measured on the planning CT images, since the inferior portion of the rectum contacted the prostate and the border was ill-defined. In addition, the rectal volume was calculated.

For each patient, calculated dosimetric parameters included the mean, minimum and maximum doses for the rectum and bladder. Additionally, the rectal volumes receiving ≥40, ≥50, ≥60, ≥65 and ≥70 Gy (rectum V_40_, V_50_, V_60_, V_65_ and V_70_, respectively) and the bladder volumes receiving ≥40 and ≥65 Gy (bladder V_40_ and V_65_, respectively) were calculated for each patient.

### Toxicity assessment

GI and GU toxicity occurring during the term of RT or <90 days after the completion of RT, were assessed according to the Common Terminology Criteria for Adverse Events version 3.0 (19). GI and GU toxicity occurring >90 days following the completion of RT were assessed according to the toxicity criteria of the Radiation Therapy Oncology Group/European Organization for Research and Treatment of Cancer acute and late radiation morbidity scoring scheme.

### Statistical analysis

Patients were classified into two groups according to BMI. The lower BMI group included patients with a BMI of <25 and the higher BMI group included those with a BMI of ≥25.

T stage and risk groups were analyzed using the G test with William’s correction. The incidences of toxicity in the two groups were assessed using a χ^2^ test. Yate’s correction was used in the assessment of late toxicity with the exception of the analysis of late GI toxicity with a cutoff value of 22.0 kg/m^2^. The patient characteristics, dosimetric parameters and anthropometric measurements were assessed using the Student’s or Welch’s t-test. Welch’s t-test was used when the variances were not equal. In addition, all statistical analyses were two-sided and P<0.05 was considered to indicate a statistically significant difference.

## Results

The mean age of the patients was 71.7 years (range, 58–84 years), the mean initial PSA level was 108.2 ng/ml (range, 4.3–2,605.0 ng/ml) and the mean prescribed dose of RT was 69.3 Gy (range, 66–70 Gy). In total, 29 patients (53.7%) had a Gleason score of ≥8. All the patients tolerated EBRT well and completed the planned course of treatment, although, one patient experienced a six day break period due to development of a spontaneous pneumothorax. The mean follow-up term was 26.7 months (range, 6–63 months) in the patients evaluated for late toxicities.

### BMI and anthropometric measurements

The lower BMI group contained 36 patients and the higher BMI group contained 18 patients. Patient characteristics are listed in Table I. No significant differences were observed in the parameters, with the exception of BMI, between the two groups. In addition, the higher BMI group included one patient with a BMI of 33.8 kg/m^2^.

The results of the anthropometric measurements are presented in Table II. The higher BMI group was found to have significantly larger abdominal circumferences and areas of total, subcutaneous and visceral fat compared with the lower BMI group. However, D was similar in the two groups and no significant differences were observed in rectal volume.

The dosimetric parameters are shown in Table III. The rectum V_60_ and V_65_ were significantly lower in the higher BMI group than in the lower BMI group, although, no significant differences were observed in the maximum doses to the PTV or rectum. In addition, no significant differences were observed in the dosimetric bladder parameters between the two groups.

### Toxicity

The results of the toxicity assessment are shown in Table IV. The incidences of acute GI and GU toxicity and late GI and GU toxicity among all the patients were 37.0, 22.2, 25.5 and 15.4%, respectively. No patients were identified with acute toxicities of grade ≥3. Late GI toxicity of grade 3 was found in five patients with BMIs of 20.1, 21.0, 21.1, 24.2 and 25.7 kg/m^2^, respectively, and GU toxicity of grade 3 was found in two patients with BMIs of 21.6 and 26.0 kg/m^2^, respectively. In addition, no patients were identified with late toxicities of grade 4.

**Figure 1:**
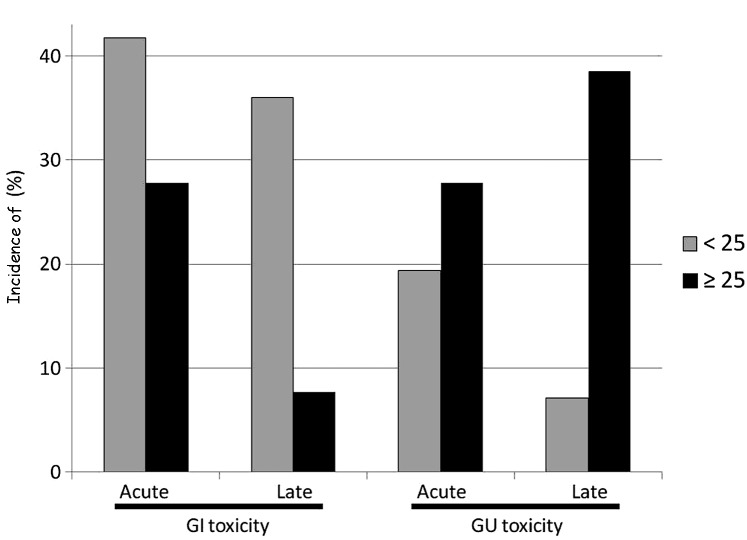
Higher BMI group exhibited a tendency to have lower incidences of acute and late GI toxicity and higher incidences of acute and late GU toxicity. No significant differences were observed between the two groups, with the exception of the incidence of late GU toxicity. Gray bars indicate the lower BMI group and black bars indicate the higher BMI group. BMI, body mass index; GI, gastrointestinal; GU, genitourinary.

The higher BMI group demonstrated lower incidences of acute and late GI toxicity and higher incidences of acute and late GU toxicity (Fig. 1). In addition, significant differences were observed between the two groups with regard to the incidence of late GU toxicity.

Multiple cutoff points were analyzed to identify an improved cutoff value for the BMI that may predict toxicity (Table V). The patients were classified into two groups according to BMI with the multiple cutoff points as follows: Patients with BMIs less than the cutoff point and patients with BMIs greater than or equal to the cutoff point. The χ^2^ test was used to calculate each P-value. Significant differences were observed when the cutoff point for the BMI was set at 22.0 and 23.0 kg/m^2^ for the late GI toxicity and a BMI of 25.0 kg/m^2^ for late GU toxicity.

## Discussion

The correlation between BMI and radiation-induced toxicity in patients with prostate cancer remains uncertain. The present study showed that patients with higher BMIs exhibited lower incidences of GI toxicity and higher incidences of GU toxicity. Previously, Wedlake *et al*(3) reported that lower BMIs predict GI toxicity in patients treated with RT for pelvic malignancies, including urological, GI and gynecological malignancies. The results of the current study are consistent with these results. However, the current study presents the anthropometric measurements and dosimetric parameters in patients with prostate cancer for two ranges of BMI. To the best of our knowledge, this is the first study to investigate the effects of BMI on the dosimetric parameters for organs at risk and radiation-induced toxicity in patients with prostate cancer.

A correlation between radiation doses and radiation-induced GI toxicity has been previously reported and appears to be well established. In addition, the risk factors for radiation-induced GI toxicity have been discussed in previous studies (1,20). However, the correlation between the BMI, radiation-induced toxicities and patient-related risk factors for toxicity in patients with prostate cancer, remain unclear. The present study examined whether BMI affects GI or GU toxicity induced by EBRT. The results showed that patients with higher BMIs have lower risks of GI toxicity and higher risks of GU toxicity.

Patil *et al*(21) previously reported that a lower BMI is associated with a higher rectal wall dose. However, a lower BMI has not been associated with greater rectal toxicity in patients with prostate cancer who are treated with brachytherapy. In the present study, higher BMIs were found to decrease the doses to the rectum and incidence of GI toxicity, although, no differences were observed in D or rectal volume. The prostate-rectum spacers, such as collagen and hydrogels, have been previously reported to separate the rectum from the prostate (22,23). The positional association between the inferior portion of the rectum and the prostate is unclear, although, the present study evaluated the area between the anterior wall of the rectum and the superior margin of the prostate. Perirectal fat may function as a spacer to reduce the rectal dose and lower the incidence of GI toxicity. The effort to reduce the rectal dose is required in patients with lower BMIs.

The higher BMI group showed higher incidences of acute and late GU toxicity, although, no apparent differences were observed between the dosimetric bladder parameters. Generally, the bladder locates sequentially to the prostate and intrapelvic fat appears to have only a small impact on the positional association between the bladder and the prostate. The bladder is a highly distensible organ. Its volume continuously changes and the post-void residual volume varies. In addition, dilation or shrinkage of the bladder wall due to pooled urine may affect the development of radiation-induced toxicity. Furthermore, fat has been reported to cause changes in the serum levels of various hormones and induce a state of chronic low-level inflammation in obese patients (8,24). These changes may affect the occurrence or severity of radiation-induced toxicity. The factors affecting GU toxicity and the biochemical mechanisms of the effects of fat on radiation-induced toxicity must be investigated in future studies.

Limitations of the current study include the sample of patients and length of the follow-up term. These limitations make it difficult to deduce specific trends. Previously, Zeleksfy *et al*(4) reported that the median time to the development of late GI and GU toxicity is 17 and 30 months, respectively, and that late GU symptoms occur significantly later than those of GI toxicity. In addition, Lawton *et al*(5) reported that the median time to the development of late GI and GU toxicity is 17.9 and 14.1 months, respectively. The median follow-up term of 26.7 months in the present study appears to be sufficient to evaluate GI and GU toxicity. With a longer follow-up period and a larger number of events, the statistical strength of the study may be reinforced.

Treatment for prostate cancer in obese patients is more challenging than that in normal-weight patients due to predictable inferior outcomes (8–14). Therefore, increased doses of EBRT with image-guided RT techniques or brachytherapy appear to have been considered for treating obese patients with prostate cancer. However, the results of the present study propose that adequate care for GU toxicity must be provided. Additional studies in larger groups of patients are required to confirm whether this is consistent with obese patients with BMIs of ≥30 kg/m^2^.

To conclude, higher BMIs tend to decrease the doses to the rectum and incidence of GI toxicity and increase the incidence of GU toxicity in patients who undergo RT to treat prostate cancer. We hypothesize that an increased effort must be made to reduce rectal doses in patients with lower BMIs. Conversely, increased care for GU toxicity must be provided for overweight patients.

## Figures and Tables

**Table I:** Patient characteristics.

	BMI, kg/m^2^		
		
Characteristics	<25.0	≥25.0	P-value
Patients, n	36	18	
Mean age, years (range)	71.7 (58–84)	71.8 (43.0–80.0)	0.97
Mean BMI, kg/m^2^ (range)	21.5 (16.1–24.6)	26.6 (25.0–33.8)	2.44×10^−10^
Clinical T stage, n (%)			0.73^a^
T1	11 (30.6)	6 (33.3)	
T2	12 (33.3)	7 (38.9)	
T3	11 (30.6)	5 (27.8)	
T4	2 (5.6)	0 (0.0)	
Bone metastasis, n (%)	7 (19.4)	2 (11.1)	0.70
Mean initial PSA, ng/ml (range)	71.9 (4.3–423.0)	180.9 (4.4–2,605.0)	0.46
Gleason score, n (%)			0.080^a^
≤6	2 (5.6)	5 (27.8)	
7	14 (38.9)	4 (22.2)	
≥8	20 (55.6)	9 (50.0)	
Risk group, n (%)			0.87^a^
Low	1 (2.8)	1 (5.6)	
Intermediate	7 (19.4)	4 (22.2)	
High	28 (77.8)	13 (72.2)	

a G test.

BMI, body mass index; PSA, prostate-specific antigen; <25.0, lower BMI group; ≥25.0, higher BMI group.

**Table II:** Anthropometric measurements.

	BMI, kg/m^2^		
		
Measurements	<25.0	≥25.0	P-value
Distance between the rectum and the prostate, mm (range)	12.1 (0.0–29.2)	10.7 (1.2–27.8)	0.48
Rectal volume, cc (range)	71.6 (35.7–151.6)	63.0 (36.6–120.4)	0.22
Bladder volume, cc (range)	79.7 (29.5–287.9)	83.4 (33.7–98.0)	0.81
Abdominal circumference, cm (range)	83.6 (64.1–105.2)	99.6 (90.3–114.1)	4.87×10^−10^
Total fat, cm^2^ (range)	183.6 (12.7–318.9)	331.6 (204.1–553.8)	4.67×10^−8^
Subcutaneous fat, cm^2^ (range)	92.6 (3.5–163.7)	170.8 (111.1–288.4)	3.23×10^−8^
Visceral fat, cm^2^ (range)	91.3 (9.2–199.9)	160.8 (63.0–287.9)	3.68×10^−5^
Ratio of visceral to subcutaneous fat, % (range)	48.9 (21.9–78.2)	47.6 (28.5–63.3)	0.67

BMI, body mass index; <25.0, lower BMI group; ≥25.0, higher BMI group.

**Table III:** Dosimetric parameters.

	BMI, kg/m^2^		
		
Parameters	<25.0	≥25.0	P-value
Mean dose for PTV (range)	69.7 (66.2–70.8)	69.0 (65.9–70.9)	0.13
Maximum dose for PTV (range)	70.9 (67.5–72.2)	70.2 (67.0–72.5)	0.15
Minimum dose for PTV (range)	65.4 (57.8–67.0)	64.9 (61.8–67.1)	0.25
Rectum			
Mean dose, Gy (range)	43.6 (29.1–58.6)	42.9 (37.3–53.2)	0.70
Maximum dose, Gy (range)	70.3 (67.1–71.8)	69.3 (66.1–72.0)	0.05
V_40_, % (range)	60.3 (34.2–92.6)	59.7 (37.2–88.7)	0.88
V_50_, % (range)	42.6 (21.1–76.4)	38.4 (20.9–67.5)	0.26
V_60_, % (range)	29.6 (12.0–57.7)	23.2 (11.1–42.0)	0.02
V_65_, % (range)	20.8 (6.5–44.5)	13.4 (3.7–31.7)	0.0049
V_70_, % (range)	3.5 (0.0–20.0)	1.4 (0.0–14.1)	0.075
Bladder			
Mean dose, Gy (range)	53.4 (30.3–64.6)	54.0 (34.2–63.5)	0.79
Maximum dose, Gy (range)	70.5 (67.2–71.7)	69.8 (66.6–72.3)	0.21
V_40_, % (range)	73.6 (36.3–94.3)	74.8 (33.7–98.8)	0.78
V_65_, % (range)	40.8 (11.3–74.3)	41.6 (2.9–68.3)	0.88

BMI, body mass index; PTV, planning target volume; <25.0, lower BMI group; ≥25.0, higher BMI group.

**Table IV:** GI and GU toxicity.

	BMI, kg/m^2^		
		
Toxicity	<25.0	≥25.0	P-value
Acute toxicity			
GI (%)	n=36	n=18	
	15 (41.7)	5 (27.8)	0.32
GU (%)	n=36	n=18	
	7 (19.4)	5 (27.8)	0.49
Late toxicity^b^			
GI (%)	n=34	n=17	
	12 (35.3)	1 (5.9)	0.053
GU (%)	n=35	n=17	
	2 (5.7)	6 (35.3)	0.018

a P-values were calculated using the χ^2^ test.

b Yate’s correction was used in the assessment of late toxicity.

BMI, body mass index; GI, gastrointestinal; GU, genitourinary; <25.0, lower BMI group; ≥25.0, higher BMI group.

**Table V:** Multiple cutoff points of the BMI for predicting GI and GU toxicity.

	BMI cut-off point, kg/m^2^						
	
Toxicity	20.0	21.0	22.0	23.0	24.0	25.0	26.0
Acute toxicity							
GI	0.89	0.97	0.35	0.73	0.48	0.49	0.94
GU	0.66	0.90	0.97	0.83	0.34	0.49	0.96
Late toxicity							
GI	0.86	0.12	0.015	0.019	0.13	0.053	0.23
GU	0.37	0.49	0.65	0.36	0.13	0.018	0.63

All values are P-values calculated by the χ^2^ test. Multiple cutoff points were examined to determine the optimal cutoff point. Yate’s correction was used in the assessment of late toxicity with the exception of the analysis of late GI toxicity with a cutoff value of 22.0 kg/m^2^. BMI, body mass index; GI, gastrointestinal; GU, genitourinary.
